# Clinical Characteristics and Immune Responses of 137 Deceased Patients With COVID-19: A Retrospective Study

**DOI:** 10.3389/fcimb.2020.595333

**Published:** 2020-12-07

**Authors:** Ning Cui, Rongdi Yan, Chunyuan Qin, Jingming Zhao

**Affiliations:** ^1^ Department of Respiratory and Critical Care Medicine, The Affiliated Hospital of Qingdao University, Qingdao, China; ^2^ School of Traditional Chinese Medicine, Tianjin University of Traditional Chinese Medicine, Tianjin, China; ^3^ Department of Occupational Disease, The Second Affiliated Hospital of Shandong University of TCM, Jinan, China; ^4^ Nuclear Medicine of Tongji Hospital Affiliated to Tongji Medical College of Huazhong University of Science and Technology, Wuhan, China

**Keywords:** COVID-19, mortality, immune responses, cytokines, lymphocyte subset

## Abstract

**Objective:**

This study aimed to evaluate the factors associated with death in patients with coronavirus disease 2019 by clarifying the clinical characteristics and immune responses.

**Methods:**

The clinical characteristics and laboratory findings, including cytokine and lymphocyte subsets, were obtained from the electronic medical records of patients in Wuhan Tongji Hospital.

**Results:**

This study included 836 patients with confirmed COVID-19. In total, 699 (83.6%) were cured and discharged, and 137 (16.4%) died. Our analysis revealed that age ≥ 65 years, male sex, malignancy, chronic obstructive pulmonary disease, dyspnea, dizziness, respiratory rate > 20 bpm, heart rate > 100 bpm, systolic blood pressure < 90 mmHg, neutrophils > 6.3×109/L, lymphopenia, thrombocytopenia, D-dimer ≥ 0.5 mg/L, lactate dehydrogenase > 250 U/L, aspartate aminotransferase > 40 U/L, total bilirubin > 26 μmol/L, albumin < 35 g/L, blood urea nitrogen > 9.5 mmol/L, estimated glomerular filtration rate < 90 ml/min/1.73, elevated cardiac troponin I, N-terminal pro-brain natriuretic peptide ≥ 900 pg/ml, C-reactive protein ≥ 25 mg/L, procalcitonin ≥ 0.05 ng/ml and ferritin > 400 μg/L were associated with death in patients with COVID-19. The multivariate logistic regression analysis revealed that an estimated glomerular filtration rate < 90 ml/min/1.73, elevated cardiac troponin I, C-reactive protein ≥ 25 mg/L and procalcitonin ≥ 0.05 ng/ml were predictive of mortality. Regarding immune responses, IL-2R, IL-6, IL-8, IL-10, and TNFα were remarkably higher in the deceased group at admission, and the levels of IL-2R, IL-6, IL-8, IL-10, and TNFα in the deceased group showed a rapid increase; the dynamics of these cytokines were highly consistent with disease deterioration. Lymphocyte subset analysis revealed that the deceased patients showed significant decreases in lymphocyte counts, especially helper T cells, suppressor T cells and NK cells.

**Conclusions:**

This study identified that an estimated glomerular filtration rate < 90 ml/min/1.73, elevated cardiac troponin I, C-reactive protein ≥ 25 mg/L and procalcitonin ≥ 0.05 ng/ml were predictors of mortality in COVID-19 patients. Elevated cytokine levels and a continued increasing trend, including in IL-2R, IL-6, IL-8, IL-10 and TNFα, and a decrease in lymphocyte subsets, especially helper T cells, suppressor T cells and NK cells, were associated with a poor prognosis.

## Introduction

A new type of coronavirus, named severe acute respiratory syndrome coronavirus 2 (SARS-CoV-2), was discovered to be the cause of unexplained pneumonia cases in Wuhan, China, in late December 2019 (Zhu et al., 2020). On February 11, 2020, the disease caused by the new coronavirus was officially named coronavirus disease 2019 (COVID-19) by the WHO. Most COVID-19 cases have mild or moderate symptoms, but severe cases can be combined with acute respiratory distress syndrome (ARDS), multiple organ dysfunction syndrome (MODS) and even death.

The World Health Organization has recently declared COVID-19 a public health emergency of international concern. Here, we performed a comprehensive exploration of the clinical features of 836 patients with confirmed COVID-19 who were transferred or admitted to the isolation ward of a Wuhan hospital. Furthermore, we evaluated 137 patients who died and 699 patients who recovered; our hope is that this study can aid clinicians in the early identification of patients with a poor prognosis.

## Methods

### Study Design and Participants

This retrospective study was conducted in Wuhan Tongji Hospital. This study was reviewed and approved by the Ethics Committee of the Affiliated Hospital of Qingdao University (Record number: QYFY WZLL 25751). All patients were diagnosed based on the Chinese Clinical Guidance for COVID-19 Pneumonia Diagnosis and Treatment (7th edition) by the National Health Commission (National Health Commission of the People’s Republic of China, 2020). When any nucleic acid test or IgG and/or IgM serology test generated a positive result, the suspected case was confirmed to be COVID-19. The enrolled patients who were confirmed to have COVID-19 were admitted to the hospital between January 14 and March 9, 2020, and all patients were discharged or deceased before March 26, 2020. The clinical outcomes (discharge, mortality, and length of stay) were monitored up to March 26, 2020, which was the final date of follow-up. Patients who were not discharged before March 26th were not included.

### Data Collection

The epidemiological, clinical, laboratory, and radiological characteristics, treatment and outcome data were obtained from electronic medical records using data collection forms. The recorded information included demographic data, medical history, exposure history, underlying comorbidities, symptoms, signs, laboratory findings, chest computed tomographic (CT) scans, and treatment measures (antiviral therapy, corticosteroid therapy, and respiratory support). The date of the disease onset was defined as the day when the first symptom was noticed.

### Statistical Analysis

The descriptive statistics included a frequency analysis (percentages) of the categorical variables and median and interquartile range (IQR) analysis of the continuous variables. Regarding the laboratory results, we also assessed whether the measurements fell within the normal range. The comparisons were performed by Student’s t-test for the continuous variables as appropriate and χ (National Health Commission of the People’s Republic of China, 2020) test for the categorical variables. Univariate and multivariate logistic regression analyses were performed to explore the association among the clinical characteristics, laboratory parameters and risk of death. The statistical significance level was set at 0.05 (two-tailed). We used SPSS (version 25.0) for all analyses.

## Results

### Demographic and Clinical Characteristics

The study population included 836 hospitalized patients with confirmed COVID-19. The median age was 64 years (range, 14–95 years), and 439 (52.5%) patients were men (**Table 1**). Of the 836 patients, 481 (57.5%) patients had at least one underlying medical condition. Hypertension (308 [36.8%]), diabetes (166 [19.9%]), coronary heart disease (86 [10.3%]), chronic obstructive pulmonary disease (48 [5.7%]), malignancy (37 [4.4%]), chronic kidney disease (41 [4.9%]), cerebrovascular disease (25 [3.0%]), chronic liver disease (22 [2.6%]) and HIV infection (2 [0.2%]) were common coexisting conditions. Ten pregnant patients and 9 medical workers all recovered and were discharged from the hospital. The median duration from onset to hospitalization was 10 days (range, 1–90 days). The most common symptoms at the onset of illness were fever (731 [87.4%]), dry cough (615 [73.6%]), dyspnea (450 [53.8%]), fatigue (352 [42.1%]), anorexia (292 [34.9%]), expectoration (288 [34.4%]), and diarrhea (244 [29.2%]). The less common symptoms included myalgia, headache, nausea, dizziness, vomiting, pharyngeal pain, abdominal pain, rhinorrhea and nasal congestion. The enrolled patients were all discharged or deceased before March 26, 2020. The median hospital stay duration was 20 days (range, 1–53 days). In total, 699 patients (83.6%) were discharged, and 137 patients (16.4%) died.

**Table 1:** Presenting demographics and clinical characteristics of deceased and recovered patients with COVID-19.

	No. (%)			*P* Value^a^
	Total (N = 836)	Deceased (n = 137)	Survivors (n = 699)	
Age, median (IOR), y	64 (51–71)	70 (62–77)	61 (48–69)	<0.001
Sex				
Female	397 (47.5)	51 (37.2)	346 (49.5)	0.009
Male	439 (52.5)	86 (62.8)	353 (50.5)	
Medical worker	9 (1.1)	0 (0.0)	9 (1.3)	0.182
Pregnancy	7 (0.8)	0 (0.0)	7 (1.0)	0.239
Underlying disease				
Hypertension	308 (76.8)	60 (43.8)	248 (35.5)	0.065
Diabetes	166 (19.9)	28 (20.4)	138 (19.7)	0.852
Coronary heart disease	86 (10.3)	20 (14.6)	66 (9.4)	0.069
Chronic obstructive pulmonary disease	48 (5.7)	16 (11.7)	32 (4.6)	0.001
Malignancy	37 (4.4)	15 (10.9)	22 (3.1)	<0.001
Gastroenteric tumor	10 (1.2)	5 (3.6)	5 (0.7)	0.076
Lung cancer	5 (0.6)	1 (0.7)	4 (0.6)	0.827
Urinary system tumors	5 (0.6)	2 (1.5)	3 (0.4)	0.331
Gynecological tumors	4 (0.5)	1 (0.7)	3 (0.4)	0.641
Hematologic malignancy	3 (0.4)	3 (2.2)	0 (0.0)	<0.001
Chronic kidney disease	41 (4.9)	9 (6.6)	32 (4.6)	0.324
Renal replacement therapy	8 (1.0)	2 (1.5)	6 (0.9)	0.508
Status post renal transplantation	5 (0.6)	1 (0.7)	4 (0.6)	0.827
Cerebrovascular disease	25 (3.0)	7 (5.1)	18 (2.6)	0.111
Chronic liver disease	22 (2.6)	3 (2.2)	19 (2.7)	0.724
HIV infection	2 (0.2)	0 (0.0)	2 (0.3)	0.531
Symptoms				
Fever	731 (87.4)	121 (88.3)	610 (87.3)	0.734
Temperature ≥ 38 °C	640 (76.6)	104 (75.9)	536 (76.7)	0.846
Temperature ≤ 38 °C	91 (10.9)	17 (12.4)	74 (10.6)	0.531
Dry cough	615 (73.6)	108 (78.8)	507 (76.5)	0.126
Dyspnea	450 (53.8)	91 (66.4)	359 (51.4)	0.001
Fatigue	352 (42.1)	50 (36.5)	302 (43.2)	0.146
Anorexia	292 (34.9)	49 (35.8)	243 (34.8)	0.822
Expectoration	288 (34.4)	46 (33.6)	242 (34.6)	0.886
Diarrhea	244 (29.2)	37 (27.0)	207 (29.6)	0.539
Myalgia	187 (22.4)	26 (19.0)	161 (23.0)	0.298
Headache	87 (10.4)	13 (9.5)	74 (10.6)	0.7
Nausea	81 (9.7)	13 (9.5)	68 (9.7)	0.931
Dizziness	73 (8.7)	19 (13.9)	54 (7.7)	0.02
Vomiting	54 (6.5)	6 (4.4)	48 (6.9)	0.279
Pharyngeal pain	43 (5.1)	2 (1.5)	41 (5.9)	0.033
Abdominal pain	25 (3.0)	7 (5.1)	18 (2.6)	0.111
Rhinorrhea	19 (2.3)	3 (2.2)	16 (2.3)	0.943
Nasal congestion	15 (1.8)	0 (0.0)	15 (2.1)	0.084
Respiratory rate, median (IQR), bpm	20 (20–24)	24 (20–32)	20 (20–22)	<0.001
Heart rate, median (IQR), bpm	91 (80–104)	100 (84–112)	90 (80–102)	<0.001
Systolic blood pressure, median (IQR), mm Hg	130 (119–145)	137 (120–151)	130 (118–143)	0.045
Diastolic blood pressure, median (IQR), mm Hg	80 (73–89)	80 (71–90)	80 (73–89)	0.586
Onset of symptom to hospitalization, median (IQR), d	10 (7–15)	10 (7–15)	10 (7–15)	0.592
Hospital length of stay, median (IQR), d	20 (14–29)	9 (5–15)	24 (17–31)	<0.001

Data are presented as medians (interquartile ranges, IQR) and n/N (%), where N is the total number of patients with available data. bpm, beats per minute; HIV, human immunodeficiency virus.

^a^P value indicate differences between deceased and recovered patients. P < 0.05 was considered statistically significant.

The deceased patients were significantly older (median age, 70 years vs 61 years) than the recovered patients. The male sex was more predominant among the deceased patients (86; 62.8%) than the recovered patients (353; 50.5%). The deceased patients were more likely to have underlying comorbidities, including chronic obstructive pulmonary disease (16 [11.7%] vs 32 [4.6%]) and malignancy (15 [10.9%] vs 22 [3.1%]). The proportion of deceased patients with hypertension, diabetes and coronary heart disease was higher, but there was no significant difference between the two groups. Compared with the recovered patients, the deceased patients were more likely to have reported dyspnea and dizziness. There were no marked differences in the median time from the onset of symptoms to hospital admission between the two groups. The median time from admission to death was 10 days, and the median time from admission to discharge was 23 days (**Table 1**).

The vital signs were measured at admission. Although the heart rates in the deceased patient group (100 beats per min (bpm)) and recovered patient group (90 bpm) were statistically significantly different, this difference was not clinically significant. Regarding the respiratory rates, both groups of patients were tachypneic, and the respiratory rates in the deceased patient group (24 bpm) were higher than those in the recovered patient group (20 bpm). The deceased patients developed tachycardia and tachypnea (respiratory rate ≥20 bpm) (49.2% and 70.4%) more often than the recovered patients (28.0% and 39.9% and 281 (33.3%)) (**Table 1**).

### Laboratory and Radiologic Findings

There were numerous abnormal results in the laboratory findings (**Table 2**), including leukopenia and lymphopenia, higher levels of C-reactive protein (CRP), erythrocyte sedimentation rate (ESR), lactate dehydrogenase and D-dimer.

**Table 2:** Presenting laboratory findings and chest CT of deceased and recovered patients with COVID-19.

	Normal Range	Median (IQR)			*P* Value^a^
		Total (N=836)	Deceased (n=199)	Survivors (n=637)	
White blood cell count, ×10^9^/L	3.5–9.5	5.7 (4.3–7.7)	9.1 (5.7–13.1)	5.4 (4.2–6.9)	<0.001
Neutrophil count, ×10^9^/L	1.8–6.3	4.1 (2.8–6.3)	8.0 (4.3–11.7)	3.9 (2.6–5.4)	<0.001
Lymphocyte count, ×10^9^/L	1.1–3.2	0.9 (0.7–1.3)	0.6 (0.4–0.8)	1.0 (0.7–1.4)	<0.001
Monocyte count, ×10^9^/L	0.1-–.6	0.4 (0.3–0.6)	0.4 (0.2–0.6)	0.4 (0.3–0.6)	0.497
Platelet count, ×10^9^/L	125–350	207 (151–280)	157 (105–224)	217 (160–291)	<0.001
Prothrombin time, s	11.5–14.5	14.1 (13.5–14.7)	15.1 (14.0–16.9)	14.0 (13.4–14.5)	<0.001
Activated partial thromboplastin time, s	29–42	39.4 (36.1–44.0)	40.2 (36.4–45.6)	39.3 (36.1–43.7)	0.038
D-dimer, mg/L	<0.5	0.98 (0.48–2.21)	2.98 (1.21–22.00)	0.76 (0.44–1.73)	<0.001
Lactate dehydrogenase, U/L	135–225	295 (229–413)	482 (371–669)	276 (222–351)	<0.001
Alanine aminotransferase, U/L	≤41	24 (16–40)	23 (16–41)	24 (15–40)	0.300
Aspartate aminotransferase, U/L	≤40	29 (20–44)	39 (28–58)	27 (19–39)	0.041
Total bilirubin, mmol/L	≤26	9.4 (7.0–12.8)	12.0 (8.9–19.0)	9.0 (6.7–11.7)	<0.001
Albumin, g/L	35–52	34.0 (30.9–37.4)	30.8 (28.0–34.0)	34.5 (31.6–37.8)	0.001
Blood urea nitrogen, mmol/L	3.1–8.0	4.5 (3.2–6.2)	8.2 (5.6–11.4)	4.2 (3.1–5.4)	<0.001
Creatinine, μmol/L	59–104	70 (57–88)	86 (66–106)	68 (57–84)	0.013
Estimated glomerular filtration rate, ml/min/1.73	>90	91 (74–103)	74 (49–91)	93 (78–104)	<0.001
Blood calcium, mmol/L	2.15–2.50	2.1 (2.1–2.2)	2.08 (1.99–2.16)	2.15 (2.07–2.23)	<0.001
**Cardiac injury**		**Total** **(N=563)**	**Deceased** **(n=115)**	**Survivors** **(n=448)**	
High-sensitivity troponin, pg/ml	≤15.6	4.7 (2.2–14.1)	30.5 (12.1–134.8)	3.6 (1.8–7.3)	0.026
N-terminal pro-brain natriuretic peptide, pg/ml	<285	178 (63–504)	853 (326–2552)	118 (51–291)	<0.001
**Inflammation-related factor**		**Total** **(N=776)**	**Deceased** **(n=126)**	**Survivors** **(n=650)**	
C-reactive protein, mg/L	<1	40.2 (9.9–88.5)	98.4 (58.4–155.1)	32.8 (8.2–75.0)	<0.001
Erythrocyte sedimentation rate, mm/h	Male<15 Female<20	37 (20–60)	37 (20–62)	37 (19–60)	0.682
Procalcitonin, ng/ml	<0.05	0.05 (0.03–0.15)	0.32(0.12–0.92)	0.04 (0.03–0.09)	0.056
Ferritin, μg/L	30–400	616 (369–1229)	1457 (763–2421)	559 (348–975)	<0.001
**Angiotensin converting enzyme**		**Total** **(N=45)**	**Deceased** **(n=6)**	**Survivors** **(n=39)**	
Angiotensin converting enzyme, U/L	8–65	24 (16.5–29.5)	20 (14–30)	24 (18–30)	0.442
**Thyroid function**		**Total** **(N=286)**	**Deceased** **(n=36)**	**Survivors** **(n=250)**	
Thyroid Stimulating Hormone, μIU/ml	0.27–4.2	1.48 (0.77–2.63)	0.69 (0.28–1.50)	1.57 (0.89–2.79)	0.121
Free triiodothyronine, pmol/L	3.1–6.8	3.91 (3.17–4.63)	2.81 (2.41–3.13)	4.04 (3.39–4.67)	<0.001
Free thyroxine, pmol/L	12–22	17.50 (14.91–19.74)	16.86 (13.42–19.35)	17.68 (15.02–19.85)	0.262
Low T3 syndrome, No.(%)		48 (16.7)	18 (50.0)	30 (11.9)	<0.001
**Co-infected with influenza**		**Total** **(N=500)**	**Deceased** **(n=79)**	**Survivors** **(n=421)**	
Co-infected with influenza A, No.(%)		223 (44.6)	31 (39.2)	192 (45.6)	0.296
Co-infected with influenza B, No.(%)		29 (3.5)	2 (2.5)	27 (6.4)	0.176
**Chest CT findings**		**Total** **(N=761)**	**Deceased** **(n=80)**	**Survivors** **(n=681)**	
Bilateral involvement		737 (96.8)	79 (98.8)	658 (96.6)	0.303
Slightly high-density shade		568 (74.6)	47 (58.8)	521 (76.5)	0.001
Ground-glass opacities		494 (64.9)	57 (71.3)	437 (64.2)	0.209
Fibrous strips		208 (27.3)	5 (6.3)	203 (29.8)	<0.001
Consolidation		113 (14.8)	8 (10.0)	105 (15.4)	0.197
Reticular appearance		49 (6.4)	2 (2.5)	47 (6.9)	0.130
Micro-nodules		15 (2.0)	0 (0.0)	15 (2.2)	0.180
Interlobular septal thickening		9 (1.2)	0 (0.0)	9 (1.3)	0.301

Data are presented as medians (interquartile ranges, IQR) and n/N (%), where N is the total number of patients with available data. CT, computed tomographic.

^a^P value indicate differences between deceased and recovered patients. P < 0.05 was considered statistically significant.

We observed substantial differences in the laboratory findings between the patients who died and those who recovered within the available data (**Table 2**). The deceased patients had more severe lymphopenia, significantly higher median neutrophil counts, and lower platelet counts. The concentrations of lactate dehydrogenase, aspartate aminotransferase, total bilirubin, blood urea nitrogen (BUN), creatinine, cardiac troponin I, N-terminal pro-brain natriuretic peptide (NT-pro-BNP), CRP, ferritin, and D-dimer were markedly higher in those who died than in those who recovered. In addition, the albumin concentrations and estimated glomerular filtration rates were significantly lower and the median prothrombin time and activated partial thromboplastin time were significantly longer in the deceased patients than in the recovered patients. There was a high proportion of patients coinfected with influenza A (223, 44.6%). The lymphocyte count and procalcitonin statistically differed between patients with and those without combined influenza A. Those without influenza A virus infection had lower lymphocyte counts and higher procalcitonin. There were no significant differences in the lymphocyte subsets and cytokine levels between the two groups (**Supplementary Material**). There was no difference in the proportion of combined influenza A between the recovered and deceased patients. >Of the 286 patients with available thyroid function data, we observed that 16.7% of these patients had low T3 syndrome, and the deceased patients had a higher incidence than the recovered patients (50% vs 11.9%) and a lower level of free triiodothyronine.

Of 761 patients with available chest CT, all patients had abnormal findings (**Table 2**). The predominant patterns of the CT findings of the patients with COVID-19 infections include slightly high-density shade (the density is between ground-glass and consolidation) (568 [74.6%]), ground-glass opacities (GGO) (494 [64.9%]), fibrous strips (208 [27.3%]), consolidation (113 [14.8%]), reticular appearance (49 [6.4%]), micronodules (15 [2.0%]) and interlobular septal thickening (9 [1.2%]). Most patients showed bilateral involvement (737 [96.8%]), and the remaining patients had unilateral involvement (right lung (21 [2.8%]) and left lung (3 [0.4%])). The extent of the lesion involvement of GGO was categorized as focal, multifocal, and diffuse related to the time of onset and severity of disease. The consolidation tended to be peripheral in distribution with lower zone predominance.

### Treatment and Outcomes

In total, 733 patients (87.7%) received oxygen therapy. In total, 157 (19.0%) patients received non-invasive ventilation (**Table 3**). Invasive mechanical ventilation was used in 64 cases (7.7%), and 6 of these cases received extracorporeal membrane oxygenation (ECMO). Almost all (765, 91.5%) patients were prescribed at least one antiviral agent, such as arborol, lopinavir/ritonavir, oseltamivir, ganciclovir, and ribavirin. Many (702, 84.0%) patients received antibacterial therapy. In total, 400 patients received systemic corticosteroid therapy (47.8%). Moreover, intravenous immunoglobin was prescribed to 216 patients (25.8%).

**Table 3:** Presenting treatment of deceased and recovered patients with COVID-19.

Treatment	No. (%)			*P* Value^a^
	Total (N=836)	Deceased (n=199)	Survivors (n=637)	
Oxygen therapy	733 (87.7)	137 (100)	600 (85.8)	<0.001
Noninvasive mechanical ventilation	157 (19.0)	114 (83.8)	43 (6.2)	<0.001
Invasive mechanical ventilation	64 (7.7)	62 (45.6)	2 (0.3)	<0.001
CRRT	2 (0.2)	2 (1.5)	0 (0.0)	0.001
ECMO	6 (0.7)	5 (3.7)	1 (0.1)	<0.001
Systemic corticosteroids therapy	400 (47.8)	105 (78.4)	295 (42.8)	<0.001
Intravenous immunoglobin	216 (25.8)	67 (49.3)	149 (21.5)	<0.001
Anti-viral drug	765 (91.5)	95 (69.3)	670 (95.9)	<0.001
Antibiotic drug	702 (84.0)	130 (94.9)	572 (81.8)	<0.001

Data are presented as n/N (%), where N is the total number of patients with available data. CRRT, continuous renal replacement therapy; ECMO, extracorporeal membrane oxygenation.

^a^P value indicate differences between deceased and recovered patients. P < 0.05 was considered statistically significant.

In total, 699 enrolled patients (83.6%) were discharged, and 137 patients (16.4%) died. More deceased patients than recovered patients received systemic corticosteroid therapy (150 [75.4%] vs 345 [40.9%]) and intravenous immunoglobulin therapy (95 [47.7%] vs 171 [20.3%]). Significantly more deceased patients received non-invasive mechanical ventilation (114 [83.8%] vs 43 [6.2%]) and invasive mechanical ventilation (62 [45.6%] vs 2 [0.3%]) (**Table 3**) than the recovered patients.

The common complications observed in the deceased patients included type I respiratory failure (106 [77.4%]), heart failure (87 [63.5%]), hyperkalemia (55 [40.1%]), acute kidney injury (52 [38%]), disseminated intravascular coagulation (44 [32.1%]), arrhythmia (29 [19.0%]), pancreas injury (21 [15.3%]), shock (15 [10.9%]) and sepsis (12 [8.8%]) (**Table 4**).

**Table 4:** Presenting complications of deceased patients with COVID-19.

Complications of deceased patients	No. (%)
	Total deceased (N=137)
Type I respiratory failure	106 (77.4)
Heart failure	87 (63.5)
Hyperkalemia	55 (40.1)
Acute kidney injury	52 (38.0)
Disseminated intravascular coagulation	44 (32.1)
Arrhythmia	26 (19.0)
Pancreas injury	21 (15.3)
Shock	15 (10.9)
Sepsis	12 (8.8)
Type II respiratory failure	7 (5.1)
Gastrointestinal bleeding	6 (4.4)
Liver failure	6 (4.4)
Pneumothorax	6 (4.4)
Alkalosis	3 (2.2)
Cerebral hemorrhage	3 (2.2)
Acute myocardial infarction	2 (1.5)
Cerebral infarction	1 (0.7)

Data are presented as N (%).

### Risk Factors for a Poor Prognosis

Of all demographic data, clinical characteristics and laboratory findings (**Tables 1** and **2**), we evaluated each variable that displayed a statistically significant difference between the deceased and recovered patients by univariate analysis. Our results showed that age ≥ 65 years, male sex, malignancy, chronic obstructive pulmonary disease, dyspnea, dizziness, respiratory rate > 20 bpm, heart rate > 100 bpm, systolic blood pressure < 90 mmHg, neutrophils > 6.3×109/L, lymphopenia, thrombocytopenia, D-dimer ≥ 0.5 mg/L, lactate dehydrogenase > 250 U/L, aspartate aminotransferase > 40 U/L, total bilirubin > 26 μmol/L, albumin < 35 g/L, blood urea nitrogen > 9.5 mmol/L, estimated glomerular filtration rate < 90 ml/min/1.73, elevated cardiac troponin I, N-terminal pro-brain natriuretic peptide ≥ 900 pg/ml, C-reactive protein ≥ 25 mg/L, procalcitonin ≥ 0.05 ng/ml and ferritin > 400 μg/L were associated with the death of patients with COVID-19 (**Table 5**). Then, we used a multivariate logistic regression model to further analyze the above variables. The model selected four variables that were predictive of mortality, including estimated glomerular filtration rate < 90 ml/min/1.73, elevated cardiac troponin I, C-reactive protein ≥ 25 mg/L and procalcitonin ≥ 0.05 ng/ml (**Table 6**).

**Table 5:** Univariate Analysis of Mortality Risk Factors for Patients with COVID-19.

Variables	Deceased	Survivors	OR (95%CI)	*P* value^a^
Age ≥ 65 y, %	69.3	42.9	3.008 (2.031–4.456)	<0.001
Male, %	62.8	50.5	1.653 (1.134–2.409)	0.009
Hypertension, %	43.8	35.5	1.417 (0.977–2.054)	0.066
Diabetes, %	20.4	19.7	1.044 (0.662–1.646)	0.852
Coronary heart disease, %	14.6	9.4	1.639 (0.958–2.807)	0.072
Chronic obstructive pulmonary disease, %	11.7	4.6	2.756 (1.467–5.178)	0.002
Malignancy, %	10.9	3.1	3.784 (1.909–7.498)	<0.001
Dyspnea, %	66.4	51.4	1.874 (1.276–2.752)	0.001
Dizziness, %	13.9	7.7	1.923 (1.100–3.362)	0.022
Respiratory rate > 20 bpm, %	70.4	39.9	3.583 (2.326–5.521)	<0.001
Heart rate > 100 bpm, %	49.2	28.0	2.486 (1.679–3.680)	<0.001
Systolic blood pressure < 90 mmHg, %	4.0	0.5	8.472 (1.998–35.926)	0.004
Neutrophil count > 6.3×10^9^/L, %	63.7	17.9	8.068 (5.403–12.048)	<0.001
Lymphocyte count < 1.1×10^9^/L, %	93.3	60.1	9.300 (4.650–18.599)	<0.001
Platelet count < 125×10^9^/L, %	34.8	9.4	5.135 (3.308–7.971)	<0.001
D-dimer ≥ 0.5 mg/L, %	95.5	68.2	9.934 (4.313–22.882)	<0.001
Lactate dehydrogenase > 250 U/L, %	90.8	62.2	6.028 (3.265–11.13)	<0.001
Aspartate aminotransferase > 40 U/L, %	47.4	24.2	2.817 (1.927–4.119)	<0.001
Total bilirubin > 26 μmol/L, %	13.3	2.3	6.510 (3.228–13.128)	<0.001
Albumin < 35 g/L, %	83.7	54.1	4.356 (2.694–7.043)	<0.001
Blood urea nitrogen > 9.5 mmol/L, %	25.9	9.5	3.325 (2.097–5.272)	<0.001
Estimated glomerular filtration rate < 90 ml/min/1.73, %	74.1	43.2	3.755 (2.484–5.677)	<0.001
Blood calcium > 2.15 mmol/L	46.6	51.6	0.816 (0.561–1.188)	0.289
Elevation of hypersensitive troponin I, %	47.0	3.3	25.554 (13.586–48.064)	<0.001
N-terminal pro-brain natriuretic peptide ≥ 450 pg/ml, %	65.3	16.4	9.563 (6.087–15.024)	<0.001
N-terminal pro-brain natriuretic peptide ≥ 900 pg/ml, %	47.5	6.3	13.517 (8.025–22.769)	<0.001
C-reactive protein ≥ 25 mg/L, %	92.9	56.0	10.214 (5.095–20.476)	<0.001
C-reactive protein ≥ 50 mg/L, %	79.4	36.8	6.614 (4.175–10.477)	<0.001
C-reactive protein ≥ 100 mg/L, %	49.2	15.7	5.205 (3.460–7.829)	<0.001
Procalcitonin ≥ 0.05 ng/ml, %	95.1	40.9	28.201 (12.223–65.066)	<0.001
Procalcitonin ≥ 0.5 ng/ml, %	35.8	4.1	13.122 (7.615–22.611)	<0.001
Ferritin > 400 μg/L, %	92.0	68.4	5.346 (2.416–11.826)	<0.001

bpm, beats per minute; CI, confidence interval; OR, odd ratio.

^a^P value indicate differences between deceased and recovered patients. P < 0.05 was considered statistically significant.

**Table 6:** Multivariate Logistic Regression Analysis of Mortality Risk Factors for Patients with COVID-19.

Variables	OR (95%CI)	*P* value^a^
Age ≥ 65 y	1.323 (0.538–3.250)	0.542
Male	1.052 (0.439–2.519)	0.909
Malignancy	1.894 (0.47––7.639)	0.369
Chronic obstructive pulmonary disease	2.212 (0.531–9.214)	0.275
Dyspnea	1.763 (0.800–3.887)	0.16
Dizziness	1.421 (0.469–4.303)	0.534
Respiratory rate > 20 bpm	1.803 (0.594–5.471)	0.078
Heart rate > 100 bpm	2.138 (0.902–5.064)	0.084
Neutrophil count > 6.3×10^9^/L	2.261 (0.983–5.203)	0.055
Lymphocyte count < 1.1×10^9^/L	1.158 (0.341–3.933)	0.814
Platelet count < 125×10^9^/L	2.435 (0.919–6.450)	0.073
D-dimer ≥ 0.5 mg/L	0.890 (0.207–3.828)	0.876
Lactate dehydrogenase > 250 U/L	0.698 (0.180–2.707)	0.603
Albumin < 35 g/L	2.613 (0.884–7.727)	0.083
Estimated glomerular filtration rate < 90 ml/min/1.73	3.859 (1.549–9.617)	0.004
Elevation of hypersensitive troponin I	4.380 (1.370–14.005)	0.013
N-terminal pro-brain natriuretic peptide ≥ 900 pg/ml	2.773 (0.997–47.054)	0.051
C-reactive protein ≥ 25 mg/L	4.391 (1.021–18.883)	0.047
Procalcitonin ≥ 0.05 ng/ml	9.676 (1.990–47.054)	0.005

bpm, beats per minute; CI, confidence interval; OR, odd ratio.

^a^P value indicate differences between deceased and recovered patients. P < 0.05 was considered statistically significant.

### Cytokines and Lymphocyte Subsets

**Figure 1:**
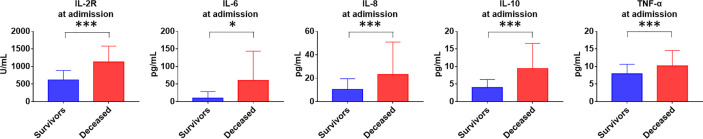
Comparison of cytokines levels at admission between deceased and recovered patients with COVID-19. IL-2R, interleukin 2 receptor; IL-6, interleukin 6; IL-8, interleukin 8; IL-10, interleukin 10; TNFα, tumor necrosis factor α. *P < .05; ***P < .001; NS, not significant.

**Figure 2:**
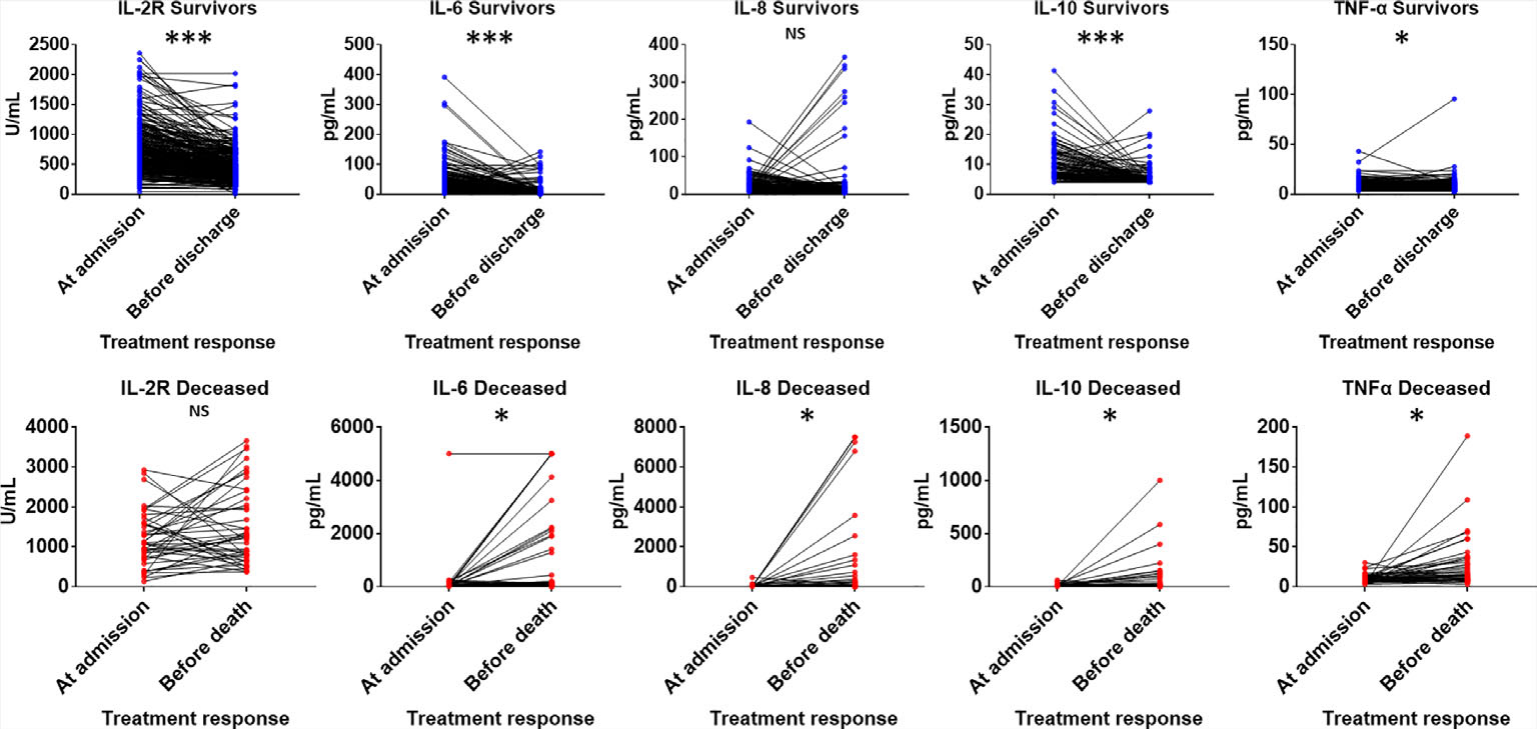
Cytokine level alterations in deceased and recovered patients with COVID-19 at admission and before discharge or death. Abbreviations: IL-2R, interleukin 2 receptor; IL-6, interleukin 6; IL-8, interleukin 8; IL-10, interleukin 10; TNFα, tumor necrosis factor α. *P < .05; ***P < .001; NS, not significant.

COVID-19 patients have high levels of circulating interleukin 2 receptor (IL-2R), IL-6, IL-10 and tumor necrosis factor α (TNFα) (**Table 7**). The levels of IL-2R, IL-6, IL-8, IL-10 and TNFα at admission in the deceased group were remarkably higher than those in the recovered group (**Figure 1**). The IL-8 levels in the deceased patients were within the normal range but showed abnormal tendencies and significantly differed from those in the recovered patients. Subsequently, the levels of IL-2R, IL-6, IL-8, IL-10 and TNFα in the deceased group showed a rapid increase during hospitalization, and the dynamics of these cytokines and related receptors were highly consistent with disease deterioration. Comparatively, the levels of IL-2R, IL-6, IL-8, IL-10 and TNFα in the recovered group showed a downward trend (**Figure 2**).

**Table 7:** Cytokines level alteration before and after treatment of patients with COVID-19.

	Normal Range	Median (IQR)			*P* Value^a^
**Cytokines level at admission**		**Total** **(N=656)**	**Deceased** **(n=94)**	**Survivors** **(n=562)**	
Interleukin 1β, pg/ml	<5	4.0 (4.0–4.0)	4.0 (4.0–4.0)	4.0 (4.0–4.0)	0.326
Interleukin 6, pg/ml	<7	12.7 (3.7–40.5)	59.8 (29.2–142.6)	9.2 (3.1–27.8)	0.022
Interleukin 8, pg/ml	<62	11.6 (6.3–21.9)	23.2 (12.9–50.8)	10.4 (5.6–19.5)	<0.001
Interleukin 10, pg/ml	<9.1	4.0 (4.0–7.6)	9.4 (4.0–16.6)	4.0 (4.0–6.3)	<0.001
Tumor necrosis factor α, pg/ml	<8.1	8.0 (5.8–10.9)	10.1 (7.2–14.6)	7.7 (5.6–10.4)	<0.001
**Cytokines level before discharge or death**		**Total** **(N=323)**	**Deceased** **(n=43)**	**Survivors** **(n=280)**	
Interleukin 6, pg/ml	<7	3.7 (1.4–11.0)	168.9 (92.4–2015.0)	3.0 (1.4–6.7)	<0.001
Interleukin 8, pg/ml	<62	8.1 (4.0–14.7)	89.4 (36.9–728.0)	7.4 (4.0–11.4)	0.002
Interleukin 10, pg/ml	<9.1	4.0 (4.0–4.0)	14.3 (5.5–73.0)	4.0 (4.0–4.0)	0.010
Tumor necrosis factor α, pg/ml	<8.1	7.5 (4.9–10.2)	14.1 (8.7–33.5)	7.0 (4.6–9.2)	<0.001
**Cytokines level alteration**		**Total** **(N=323)**	**Deceased** **(n=43)**	**Survivors** **(n=280)**	
Interleukin 6, pg/ml		−5.5 (−28.8/0.0)	97.0 (−1.6/1778.3)	−7.5 (−30.0/−0.9)	<0.001
Interleukin 8, pg/ml		−1.4 (−11.0/3.5)	60.8 (11.3/633.5)	−2.3 (−11.8/−0.9)	0.003
Interleukin 10, pg/ml		0.0 (−2.0/0.0)	5.9 (−2.0/52.3)	0.0 (−2.1/0.0)	0.017
Tumor necrosis factor α, pg/ml		−0.7 (−3.1/1.6)	2.9 (−0.3/25.3)	−0.9 (−3.3/1.3)	0.002

Data are presented as medians (interquartile ranges, IQR).

^a^P value indicate differences between deceased and recovered patients. P < 0.05 was considered statistically significant.

**Figure 3:**
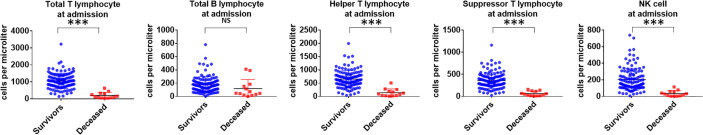
Comparison of peripheral lymphocyte subset levels at admission between deceased and recovered patients with COVID-19. ***P < .001; NS, not significant.

In our study, lymphopenia was common in the patients with COVID-19 (65.5%), indicating an impairment of the immune system during the course of infection. We further analyzed different subsets of lymphocytes. Decreases in the total number of T cells (CD3+CD19-), helper T cells (CD3+CD4+), suppressor T cells (CD3+CD8+) and NK cells (CD3−/CD16+CD56+) were also observed in the COVID-19 patients (**Table 8**). We found that compared to the recovered patients, the deceased patients showed significant decreases in lymphocyte subsets, especially helper T cells, suppressor T cells and NK cells (**Figure 3**). The ratio of helper T and suppressor T (Th/Ts) cells was still within the normal range and did not differ between the two groups. Furthermore, there was no significant difference between the two groups in B cells (CD3-CD19+). This finding indicates that T lymphocytes were more suppressed in severe patients than B cells and NK cells and that SARS-CoV-2 has a negative impact on T-cell mediated immunity. The same difference was also observed in the lymphocyte subsets before discharge or before death; however, the alteration in the lymphocyte subsets between the two groups before and after treatment was not significant.

**Table 8:** Peripheral lymphocyte subset alteration before and after treatment of patients with COVID-19.

	Normal Range	Median (IQR)			*P* Value^a^
**Lymphocyte subsets at admission**		**Total** **(N=131)**	**Deceased** **(n=13)**	**Survivors** **(n=118)**	
Total T lymphocyte (CD3+CD19-), %	50–84	73.7 (64.7–80.1)	54.4 (39.5–74.0)	73.9 (65.6–80.5)	0.628
Total T lymphocyte (CD3+CD19-), per microliter	955–2860	977 (654–1280)	122 (57–322)	1047 (760–1330)	<0.001
Total B lymphocyte (CD3-CD19+), %	5–18	12.39 (8.1–17.2)	27.1 (17.7–44.4)	11.8 (8.0–16.1)	0.866
Total B lymphocyte (CD3-CD19+, per microliter	90–560	155 (84–218)	48 (25–168)	161 (92–226)	0.096
Helper T lymphocyte (CD3+CD4+), %	27–51	43.7 (36.3–49.8)	29.5 (21.6–53.5)	44.0 (37.4–49.8)	0.614
Helper T lymphocyte (CD3+CD4+), per microliter	550–1440	561 (358–796)	93 (30–225)	610 (446–808)	<0.001
Suppressor T lymphocyte (CD3+CD8+), %	15–44	23.5 (18.0–30.6)	13.5 (10.2–21.8)	24.1 (19.2–31.3)	0.556
Suppressor T lymphocyte (CD3+CD8+), per microliter	320–1250	305 (182–455)	51 (17–122)	336 (245–449)	<0.001
NK cell (CD3-/CD16+CD56+), %	7–40	11.9 (7.9–18.6)	8.1 (3.7–16.4)	12.7 (8.0–18.7)	0.624
NK cell (CD3-/CD16+CD56+), per microliter	150–1100	146 (103–246)	28 (5–60)	167 (112–252)	<0.001
Th/Ts	0.71–2.78	1.95 (1.36–2.55)	2.01 (1.32–4.04)	1.88 (1.36–2.52)	0.285
**lymphocyte subsets before discharge or death**		**Total** **(N=59)**	**Deceased** **(n=5)**	**Survivors** **(n=54)**	
Total T lymphocyte (CD3+CD19-), per microliter	955–2860	977 (654–1370)	221 (141–427)	1100 (843–1423)	<0.001
Total B lymphocyte (CD3-CD19+, per microliter	90–560	137 (85–210)	61 (29–143)	144 (86–212)	0.074
Helper/induced T lymphocyte (CD3+CD4+), per microliter	550–1440	610 (358–830)	189 (113–319)	631 (409–840)	0.003
Suppressor T lymphocyte (CD3+CD8+), per microliter	320–1250	338 (167–511)	29 (25–98)	350 (254–525)	<0.001
NK cell (CD3-/CD16+CD56+), per microliter	150–1100	176 (119–288)	17 (12–63)	194 (129–293)	0.012
Th/Ts	0.71–2.78	1.91 (1.35–2.53)	4.87 (2.24–6.69)	1.77 (1.32–2.28)	0.901
**lymphocyte subsets alteration**		**Total** **(N=59)**	**Deceased** **(n=5)**	**Survivors** **(n=54)**	
Total T lymphocyte (CD3+CD19-), per microliter		123 (−53/222)	−9 (−194/141)	127 (−49/232)	0.394
Total B lymphocyte (CD3-CD19+, per microliter		−6 (−24/21)	−8 (−144 to 21)	−4 (−23/22)	0.268
Helper/induced T lymphocyte (CD3+CD4+), per microliter		71 (−16/120)	75 (−173/130)	68 (−15/120)	0.909
Suppressor T lymphocyte (CD3+CD8+), per microliter		31 (−13/108)	8 (−67/14)	40 (−9/109)	0.245
NK cell (CD3-/CD16+CD56+), per microliter		7 (−44/78)	−48 (−65/25)	13 (−42/85)	0.391

Data are presented as medians (interquartile ranges, IQR). Th/Ts, the ratio of helper T lymphocyte and suppressor T lymphocyte.

^a^P value indicate differences between deceased and recovered patients. P < 0.05 was considered statistically significant.

## Discussion

In this retrospective study, we report 836 patients confirmed to have COVID-19 and provide the detailed clinical characteristics of this cohort of patients, including 137 fatal cases. We further comprehensively describe the major differences in the clinical features and immune responses between the deceased patients and those who recovered. We hope that this study could help clinicians identify patients with a poor prognosis early by increasing awareness of some characteristics indicative of a higher risk, realizing effective patient risk stratification and helping appropriately deploy health care resources.

We found that older age, male sex, baseline diseases such as hematological and neoplastic disorders and COPD, the presence of dyspnea and dizziness, deterioration of vital signs, evidence of increased acute inflammation and end organ damage (cardiac, renal, liver, thyroid and hematologic) at admission were associated with an increased risk of mortality due to COVID-19 infection, which is consistent with the results of previous clinical studies (Tian et al., 2020). The multivariate analysis showed that a glomerular filtration rate < 90 ml/min/1.73, elevated cardiac troponin I, C-reactive protein ≥ 25 mg/L and procalcitonin ≥ 0.05 ng/ml were predictors of a poor prognosis. We recommend that clinicians closely monitor renal function, cardiac troponin I, C-reactive protein and procalcitonin as markers of potential progression to critical illness. The troponin values could be elevated in patients with renal disease in the absence of cardiac injury. In our study, the proportion of renal insufficiency in the patients with elevated troponin was higher than that in the patients with normal troponin (8 [11.6%] vs 20 [4.0%]); however, there was no statistically significant difference.

We found a decreased estimated glomerular filtration rate in 48.2% of the COVID-19 patients, and this finding is strongly associated with mortality despite the significant correlation between chronic kidney disease and poor prognosis in our analysis. We found that 36.6% of the deceased patients developed acute kidney injury (AKI) during hospitalization. The etiology of AKI in COVID-19 cases has not been fully elucidated (Hirsch et al., 2020). The close relationship between AKI and the occurrence of respiratory failure implies, to some extent, ischemic acute tubular necrosis, which is consistent with the autopsy results of 26 patients who presented with renal tubular injury as the primary renal finding (Su et al., 2020). However, due to the lack of the baseline CKD status, we cannot specifically consider this factor a risk factor.

Although we did not find that coronary heart disease is associated with a poor prognosis, other reports have shown that cardiovascular disease may increase susceptibility to COVID-19 and its severity (Mehra et al., 2020). However, our results showed elevated cardiac biomarkers, arrhythmia and heart failure, even in the patients without pre-existing cardiovascular disease, suggesting that myocardial injury is an important pathogenic feature of COVID-19. Furthermore, we found that elevated cardiac troponin was an indicator of a poor prognosis. The exact mechanism by which SARS-CoV-2 causes myocardial injury is still unclear. The proposed mechanisms of myocardial injury include direct damage to cardiomyocytes, systemic inflammation, myocardial interstitial fibrosis, interferon-mediated immune response, excessive cytokine response of lymphocytes, coronary plaque instability and hypoxia (Babapoor-Farrokhran et al., 2020).

Coinfection with SARS-CoV-2 and other viruses has been described in other studies, but the reported frequency is variable ranging from 0.02% to 2.6% (Ding et al., 2020; Richardson et al., 2020; Kim et al., 2020). In our study, there was a higher proportion of patients coinfected with influenza A (223, 44.6%). This finding may be related to the overlap between this research phase and the high-incidence period of influenza A in the local area. Additionally, the clinical characteristics and immune response of the patients coinfected with both COVID-19 and influenza virus were similar to those of the COVID-19 cases. There was no significant difference in the rates of coinfection with influenza A between the recovered and deceased patients. The coinfection with influenza A did not affect the prognosis of the patients with COVID-19.

In addition, SARS-CoV-2 can infect host cells by interacting with membrane-bound angiotensin-converting enzyme 2 (ACE2) in cardiomyocytes (Reynolds et al., 2020). Treatment with renin-angiotensin-aldosterone system (RAAS) inhibitors can increase the tissue expression of ACE2 and its presentation at the cytomembrane, increasing the risk and severity of COVID-19 infection (Vaduganathan et al., 2020). Therefore, discontinuation or alternative antihypertensive drugs have been suggested for COVID-19 patients treated with ACE inhibitors or angiotensin-receptor blockers (ARBs). We found no significant difference in the plasma ACE concentrations between the deceased group and the recovered group. However, the results were restricted by the small number of samples; furthermore, we did not test the AEC2 concentration or previous medication history of AEC inhibitors or ARBs.

Mixed bacterial infection is an important contributor to mortality during the COVID-19 epidemic period (Hendaus and Jomha, 2020). The multivariate analysis revealed that the concentration of procalcitonin (PCT) and C-reactive protein (CRP) at admission remained robust in the adverse outcome prediction. Patients with C-reactive protein ≥ 25 mg/L and procalcitonin ≥ 0.05 ng/ml have a greater than 4-fold and nearly 10-fold higher risk of mortality, respectively. However, our inclusion criteria allowed patients with chronic diseases, which can directly affect the CRP and PCT levels (such as malignancy and renal dysfunction). Further analysis of the kinetics of CRP, PCT and other inflammatory biomarkers may play a role in predicting evolution. The rate of concurrent bacterial infections in COVID-19 patients is unclear. Nonetheless, we observed that a substantial proportion of the patients received empirical antimicrobial treatment. Therefore, it is crucial and urgent to standardize antibiotic application.

Most patients with COVID-19 exhibit substantially elevated serum levels of pro-inflammatory cytokines, including IL-6, IL-2R, IL-8, IL-10 and TNFα, which is characterized as cytokine storms. High levels of pro-inflammatory cytokines may lead to shock and tissue damage to the lungs, heart, liver and kidneys, which can be confirmed by our data. These increases lead to massive infiltration of neutrophils and macrophages, diffuse alveolar damage with the formation of hyaline membranes and diffuse thickening of the alveolar wall (Cao, 2020).

Lymphocyte subsets play an important role in cellular immune regulation (Wan et al., 2020). We found that helper T cells and suppressor T cells showed greater reductions in the deceased group than in the recovered group. This finding suggests that T lymphocytes provide an important defense against COVID-19 and that SARS-CoV-2 has a negative impact on T-cell mediated immunity, which is consistent with infections with MERS coronavirus and SARS coronavirus (Wong et al., 2003; Chu et al., 2016). T lymphocytes and their subsets might be a potential predictor of disease severity and clinical efficacy in COVID-19.

COVID-19 is associated with both immunodeficiency and hyperinflammation, and T cell numbers are negatively correlated with serum IL-6, IL-10 and TNF-α. As the virus can directly infect T cells, T cell infection may result in cell death by apoptosis or necrosis (Yue et al., 2018; Jamilloux et al., 2020). In addition, immune cell redistribution due to the accumulation of lymphocytes in the lungs may lead to lymphopenia (Sarzi-Puttini et al., 2020). T cell exhaustion may stem from increased programmed death 1 (PD-1) or T cell immunoglobulin and mucin domain-3 (Tim-3) expression, elevated levels of inhibitory IL-10 (Diao et al., 2020) or increased IL-6-induced expression of suppressor of cytokine signaling (SOCS3) (Croker et al., 2003). Moreover, increased IL-2 and IL-7, which are the cytokines responsible for the expansion and differentiation of T cell subsets, may attempt to reverse lymphopenia and T cell exhaustion (Huang et al., 2020).

Studies have shown that type-I interferon (IFN) is critical for preventing viral replication and triggers subsequent impairment and hyperactivation of the immune system, including T cell exhaustion and cytokine storm (Jamilloux et al., 2020). Treatments should focus on reducing the viral load using antivirals and immune boosters that stimulate type-I IFN promptly at symptom onset while targeting pro-inflammatory cytokines with immunosuppressants at the start of the cytokine storm. Therefore, starting antiviral treatment as soon as possible during the very early stage of the disease and closely monitoring the cytokine levels are critical for managing treatment strategies.

We identified many candidate variables for risk stratification that may serve as clinical predictors of fatal COVID-19 and explored the immunodeficiency and hyperinflammation state during the course of COVID-19. Our research has some limitations, and some potential bias may arise. First, this study was a retrospective and single-center study, and some data were missing; a larger cohort study should be conducted to evaluate the immune response after COVID-19 infection. Second, the patients diagnosed with COVID-19, especially those with severe cases and bacterial coinfection, might affect the results of the immune response. Most patients presented increased procalcitonin, which was more evident in the severe cases. Despite these limitations, our study demonstrated several indicators suggesting that because of the dysregulated immune response in COVID-19 patients, SARS-CoV-2 might mainly act on T lymphocytes, inducing a cytokine storm in the body and generating inflammatory damage; thus, surveillance of lymphocyte subsets and serum levels of pro-inflammatory cytokines is helpful for the early screening of critical cases, risk stratification and management strategy adjustment.

## Conclusions

To evaluate the factors associated with death in COVID-19 patients, we collected clinical characteristics and laboratory findings, including cytokines and lymphocyte subsets, from 836 laboratory confirmed patients, including 137 deceased patients and 699 survivors. In conclusion, this study identified four predictors, i.e., an estimated glomerular filtration rate < 90 ml/min/1.73, elevated cardiac troponin I, C-reactive protein ≥ 25 mg/L and procalcitonin ≥ 0.05 ng/ml, of mortality among COVID-19 patients. We further found that elevated and a trend of a continued increase in cytokine levels, including IL-2R, IL-6, IL-8, IL-10 and TNFα, and decreased lymphocyte subsets, especially helper T cells, suppressor T cells and NK cells, were associated with a poor prognosis of COVID-19. Hence, paying attention to patients with renal insufficiency, myocardial injury, bacterial co-infection, impaired T-cell immune function, and dynamic increase in cytokines could help clinicians identify patients with a poor prognosis.

## Data Availability Statement

The original contributions presented in the study are included in the article/**Supplementary Material**. Further inquiries can be directed to the corresponding authors.

## Ethics Statement

The study was reviewed and approved by the Ethics Committee of the Affiliated Hospital of Qingdao University (Record number: QYFY WZLL 25751). Written informed consent from the participants was not required to participate in this study in accordance with the national legislation and the institutional requirements.

## Author contributions

RY, CQ, and JZ collected the epidemiological and clinical data. NC and JZ summarized all the data. NC and RY drafted the manuscript. CQ and JZ revised the final manuscript. All authors contributed to the article and approved the submitted version.

## Funding

This work was supported by the Medicine and Health Technology Development Plan Project of Shandong Province (Grant NO. 2019WS377), Traditional Chinese Medicine Research Project of Qingdao City (Grant NO. 2020-zyy059), and the Affiliated Hospital of Qingdao University Youth Research Fund (Grant NO. 2019030).

## Conflict of Interest

The authors declare that the research was conducted in the absence of any commercial or financial relationships that could be construed as a potential conflict of interest.

## Abbreviations

COVID-19, coronavirus disease 2019; SARS-CoV-2, severe acute respiratory syndrome coronavirus 2; ARDS, acute respiratory distress syndrome; MODS, multiple organ dysfunction syndrome; CT, computed tomographic; IQR, interquartile range; CRP, C-reactive protein; ESR, erythrocyte sedimentation rate; BUN, blood urea nitrogen; NT-pro-BNP, N-terminal pro-brain natriuretic peptide; GGO, ground-glass opacities; ECMO, extracorporeal membrane oxygenation; IL-2R, interleukin 2 receptor; TNFα, tumor necrosis factor α; AKI, acute kidney injury; ACE2, angiotensin-converting enzyme 2; RAAS, renin-angiotensin-aldosterone system; ARBs, angiotensin-receptor blockers; PCT, procalcitonin; PD-1, programmed death 1; Tim-3, T cell immunoglobulin and mucin domain-3; SOCS3, suppressor of cytokine signaling; IFN, interferon.
