# Supplementary material for: Clinical Characteristics and Immune Responses of 137 Deceased Patients With COVID-19: A Retrospective Study
Source: Front Cell Infect Microbiol. 2020 Dec 7;10:595333. doi: 10.3389/fcimb.2020.595333 (PMC7750484; doi:10.3389/fcimb.2020.595333)
Supplement: Supplementary file 1 [file Table_1.docx]

Supplement table 1 Presenting relevant laboratory findings, cytokines level alteration and peripheral lymphocyte subset alteration of COVID-19 patients co-infected with and without influenza A.

Data are presented as medians (interquartile ranges, IQR).

^a^ P value indicate differences between deceased and recovered patients. P < 0.05 was considered statistically significant.

|  | **Normal**  **Range** | **Median (IQR)** | | | ***P* Value^a^** |  |
| --- | --- | --- | --- | --- | --- | --- |
|  |  | **Total**  **(N=500)** | **Co-infected with influenza A**  **(n=223)** | **Co-infected without influenza A**  **(n=277)** |  |  |
| White blood cell count, ×10^9^/L | 3.5-9.5 | 5.8(4.4-7.7) | 5.8(4.4-7.7) | 5.7(4.5-7.6) | 0.354 |  |
| Lymphocyte count, ×10^9^/L | 1.1-3.2 | 0.9(0.6-1.3) | 1.0(0.7-1.4) | 0.9(0.6-1.3) | 0.045 |  |
| **Cardiac injury** |  | **Total**  **(N=358)** | **Co-infected with influenza A**  **(n=159)** | **Co-infected without influenza A**  **(n=199)** |  |  |
| High-sensitivity troponin, pg/mL | ≤15.6 | 4.9(2.4-12.5) | 3.8(2.0-9.3) | 5.7(2.8-15.9) | 0.599 |  |
| N-terminal pro-brain natriuretic peptide, pg/mL | ＜285 | 163(61-410) | 157(46-385) | 170(66-425) | 0.068 |  |
| **Inflammation-related factor** |  | **Total**  **(N=429)** | **Co-infected with influenza A**  **(n=196)** | **Co-infected without influenza A**  **(n=233)** |  |  |
| C-reactive protein, mg/L | ＜1 | 40.9(9.6-91.6) | 38.8(7.0-86.2) | 44.2(11.2-102.9) | 0.069 |  |
| Erythrocyte sedimentation rate, mm/h | Male＜15  Female＜20 | 36(19-60) | 36(16-63) | 35(21-58) | 0.883 |  |
| Procalcitonin, ng/mL | ＜0.05 | 0.06(0.03-0.14) | 0.05(0.03-0.12) | 0.06(0.03-0.15) | 0.018 |  |
| Ferritin, μg/L | 30-400 | 651(379-1305) | 605(360-1173) | 727(387-1351) | 0.798 |  |
| **C****ytokines level at admission** |  | **Total**  **(N=404)** | **Co-infected with influenza A**  **(n=182)** | **Co-infected without influenza A**  **(n=222)** |  |  |
| Interleukin 1β, pg/mL | ＜5 | 4.0(4.0-4.0) | 4.0(4.0-4.0) | 4.0(4.0-4.0) | 0.902 |  |
| Interleukin 6, pg/mL | ＜7 | 13.3(4.0-42.4) | 14.2(3.8-47.1) | 13.2(4.6-40.8) | 0.845 |  |
| Interleukin 8, pg/mL | ＜62 | 12.3(7.0-23.4) | 10.8(5.7-20.4) | 14.0(7.8-25.9) | 0.302 |  |
| Interleukin 10, pg/mL | ＜9.1 | 4.0(4.0-8.2) | 4.0(4.0-8.3) | 4.0(4.0-8.0) | 0.473 |  |
| Tumor necrosis factor α, pg/mL | ＜8.1 | 8.1(6.1-11.2) | 8.1(6.0-10.7) | 8.3(6.1-11.6) | 0.531 |  |
| **Cytokines level before discharge or death** |  | **Total**  **(N=205)** | **Co-infected with influenza A**  **(n=86)** | **Co-infected without influenza A**  **(n=119)** |  |  |
| Interleukin 6, pg/mL | ＜7 | 3.8(1.4-10.7) | 3.1(1.4-8.1) | 4.5(1.4-12.9) | 0.141 |  |
| Interleukin 8, pg/mL | ＜62 | 8.2(4.0-15.2) | 8.0(4.0-12.2) | 8.7(5.2-21.4) | 0.380 |  |
| Interleukin 10, pg/mL | ＜9.1 | 4.0(4.0-4.0) | 4.0(4.0-4.0) | 4.0(4.0-5.1) | 0.977 |  |
| Tumor necrosis factor α, pg/mL | ＜8.1 | 7.6(5.0-10.3) | 7.5(4.9-9.7) | 7.6(5.1-10.4) | 0.987 |  |
| **Cytokines level alteration** |  | **Total**  **(N=205)** | **Co-infected with influenza A**  **(n=86)** | **Co-infected without influenza A**  **(n=119)** |  |  |
| Interleukin 6, pg/mL |  | -5.5(-33.6/0.0) | -12.5(-45.9/0.0) | -7.5(-30.0/-0.9) | 0.262 |  |
| Interleukin 8, pg/mL |  | -2.2(-11.8/3.2) | -1.5(-11.1/2.2) | -2.3(-11.8/-0.9) | 0.436 |  |
| Interleukin 10, pg/mL |  | 0.0(-2.3/0.0) | 0.0(-3.3/0.0) | 0.0(-2.1/0.0) | 0.984 |  |
| Tumor necrosis factor α, pg/mL |  | -0.9(-3.3/1.6) | -1.2(-3.5/1.4) | -0.9(-3.3/1.3) | 0.918 |  |
| **lymphocyte subsets at admission** | |  | **Total**  **(N=87)** | **Co-infected with**  **influenza A (n=43)** | **Co-infected without**  **influenza A (n=44)** |  |
| Total T lymphocyte (CD3+CD19-), % | | 50-84 | 73.8(64.8-78.4) | 73.9(67.5-79.6) | 73.4(59.0-78.1) | 0.392 |
| Total T lymphocyte (CD3+CD19-), per microliter | | 955-2860 | 1047(650-1361) | 1073(705-1409) | 1008(549-1250) | 0.487 |
| Total B lymphocyte (CD3-CD19+), % | | 5-18 | 12.8(9.5-17.8) | 11.7(7.5-15.8) | 15.0(10.2-22.0) | 0.899 |
| Total B lymphocyte (CD3-CD19+ , per microliter | | 90-560 | 170(87-226) | 151(72-209) | 180(91-231) | 0.387 |
| Helper T lymphocyte (CD3+CD4+), % | | 27-51 | 45.7(39.0-52.4) | 43.7(37.5-53.5) | 46.5(40.0-49.9) | 0.319 |
| Helper T lymphocyte (CD3+CD4+), per microliter | | 550-1440 | 619(360-859) | 616(415-884) | 622(330-814) | 0.962 |
| Suppressor T lymphocyte (CD3+CD8+), % | | 15-44 | 22.8(17.3-30.2) | 24.2(18.6-32.4) | 21.2(15.6-26.1) | 0.992 |
| Suppressor T lymphocyte (CD3+CD8+), per microliter | | 320-1250 | 315(178-446) | 335(233-460) | 289(2149-421) | 0.206 |
| NK cell (CD3-/CD16+CD56+), % | | 7-40 | 11.0(7.3-17.8) | 12.8(7.3-18.7) | 10.1(7.3-15.3) | 0.269 |
| NK cell (CD3-/CD16+CD56+), per microliter | | 150-1100 | 139(98-240) | 162(101-288) | 135(87-214) | 0.315 |
| Th/Ts | | 0.71-2.78 | 2.01(1.51-2.70) | 2.02(1.36-2.69) | 2.01(1.66-2.82) | 0.210 |
| **lymphocyte subsets before discharge or death** | |  | **Total**  **(N=50)** | **Co-infected with**  **influenza A (n=25)** | **Co-infected**  **without influenza A (n=25)** |  |
| Total T lymphocyte (CD3+CD19-), per microliter | | 955-2860 | 1050(616-1383) | 1106(791-1262) | 995(572-1472) | 0.912 |
| Total B lymphocyte (CD3-CD19+ , per microliter | | 90-560 | 145(86-212) | 117(67-192) | 175(104-218) | 0.367 |
| Helper/induced T lymphocyte (CD3+CD4+), per microliter | | 550-1440 | 617(356-832) | 640(376-817) | 586(325-844) | 0.687 |
| Suppressor T lymphocyte (CD3+CD8+), per microliter | | 320-1250 | 320(192-525) | 301(230-463) | 359(157-553) | 0.935 |
| NK cell (CD3-/CD16+CD56+), per microliter | | 150-1100 | 189(123-307) | 176(128-317) | 192(110-297) | 0.709 |
| Th/Ts | | 0.71-2.78 | 1.96(1.35-2.56) | 1.96(1.32-2.36) | 1.98(1.35-3.56) | 0.300 |
| **lymphocyte subsets alteration** | |  | **Total**  **(N=50)** | **Co-infected with**  **influenza A (n=25)** | **Co-infected**  **without influenza A (n=25)** |  |
| Total T lymphocyte (CD3+CD19-), per microliter | |  | 121(-49/212) | 95(-121/196) | 124(-13/225) | 0.791 |
| Total B lymphocyte (CD3-CD19+ , per microliter | |  | -7(-33/17) | -13(-40-18) | 0(-26/19) | 0.955 |
| Helper/induced T lymphocyte (CD3+CD4+), per microliter | |  | 68(-42/109) | 58(-87/110) | 80(-16/118) | 0.887 |
| Suppressor T lymphocyte (CD3+CD8+), per microliter | |  | 35(-2/109) | 43(-1.5/109) | 21(-5/110) | 0.580 |
| NK cell (CD3-/CD16+CD56+), per microliter | |  | 13(-42/85) | 36(-44/85) | 7(-37/105) | 0.291 |
